# Modified Method of rRNA Structure Analysis Reveals Novel Characteristics of Box C/D RNA Analogues

**Published:** 2015

**Authors:** J. A. Filippova, G. A. Stepanov, D. V. Semenov, O. A. Koval, E. V. Kuligina, I. V. Rabinov, V. A. Richter

**Affiliations:** Institute of Chemical Biology and Fundamental Medicine, Siberian Branch of the Russian Academy of Sciences, Lavrentiev Ave., 8, Novosibirsk, 630090, Russia; Novosibirsk State University, Pirogova Str., 2, Novosibirsk, 630090, Russia

**Keywords:** small nucleolar box C/D RNAs, RNA post-transcriptional modifications, RNA 2’-O-methylation, reverse transcription termination

## Abstract

Ribosomal RNA (rRNA) maturation is a complex process that involves chemical modifications of the bases or sugar residues of specific nucleotides. One of the most abundant types of rRNA modifications, ribose 2’-O-methylation, is guided by ribonucleoprotein complexes containing small nucleolar box C/D RNAs. Since the majority of 2’-O-methylated nucleotides are located in the most conserved regions of rRNA that comprise functionally important centers of the ribosome, an alteration in a 2’-O-methylation profile can affect ribosome assembly and function. One of the key approaches for localization of 2’-O-methylated nucleotides in long RNAs is a method based on the termination of reverse transcription. The current study presents an adaptation of this method for the use of fluorescently labeled primers and analysis of termination products by capillary gel electrophoresis on an automated genetic analyzer. The developed approach allowed us to analyze the influence of the synthetic analogues of box C/D RNAs on post-transcriptional modifications of human 28S rRNA in MCF-7 cells. It has been established that the transfection of MCF-7 cells with a box C/D RNA analogue leads to an enhanced modification level of certain native sites of 2’-O-methylation in the target rRNA. The observed effect of synthetic RNAs on the 2’-O-methylation of rRNA in human cells demonstrates a path towards targeted regulation of rRNA post-transcriptional maturation. The described approach can be applied in the development of novel diagnostic methods for detecting diseases in humans.

## INTRODUCTION

The RNAs of all living organisms undergo post-transcriptional modifications and contain not only the canonical, but also the modified nucleotides necessary for the proper functioning of sophisticated biological complexes. Post-transcriptional modifications significantly influence the formation of the RNA secondary structure and function [1, 2].

One of the most abundant types of non-coding RNA modifications in mammals is the 2’-O-ribose methylation of nucleotides [3]. The position of many 2’-O-methylation sites in rRNA is conserved, and the majority of the modifications have been found in most evolutionary- conserved and functionally important regions of rRNA that play the key role in different stages of translation [4]. A change in the overall pattern of rRNA methylation and the lack of modified nucleotides can result in aberrations in ribosome assembly and function [5, 6]. For instance, it has been demonstrated that the blockage of nucleotide modification in the ribosomal peptidyl transferase center (PTC) causes changes in the secondary structure of 25S rRNA in yeast, impairs the translational activity of ribosomes and, in some cases, enhances cellular sensitivity to translational inhibitors [2, 7].

Modifications are considered to promote the stabilization of the rRNA functional structure due to the influence on inter- and intramolecular interactions [2]. So far, the main function of modifications is believed to be participation in ribosome maturation and assembly: rRNA modification may serve to some extent as an additional quality criterion of newly synthesized rRNA, contributing to the selection of only ‘’proper’’ rRNAs for integration into ribosomes [8].

**Fig. 1:**
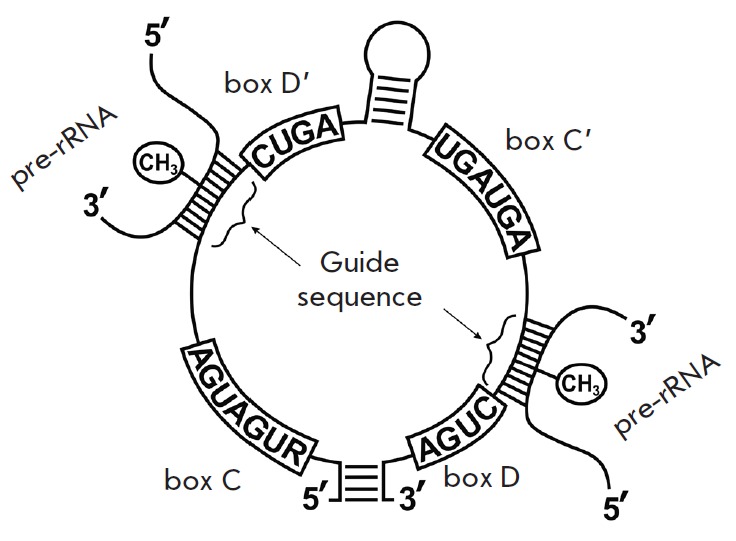
Structure of box C/D RNA and its interaction with a target pre-rRNA

Ribose 2’-O-methylation of rRNAs and small nuclear RNAs in mammalian cells is known to be performed by ribonucleoprotein complexes containing a small nucleolar box C/D RNA (snoRNPs) [9]. Furthermore, box C/D RNA, being complementary to the region of the target RNA, is directly involved in the process of recognition of the target nucleotide for 2’-O-methylation; the fifth nucleotide from the box D is subjected to modification *(Fig. 1)* [10, 11].

It has been recently shown that several types of cancer cells exhibit enhanced expression of box C/D RNAs, as well as of fibrillarin, the key protein of snoRNPs, which functions as a methyltransferase [12, 13]. It has been established that various types of breast cancer cells can differ in the methylation level of several rRNA nucleotides [14]. Differences in the expression of certain box C/D RNAs have been detected in various types of leukemia [15]. It has been mentioned earlier that aberrations in rRNA maturation and ribosomal protein synthesis in human cells can result in pathological changes [16, 17]. Thus, snoRNAs are able to participate in oncogenesis, and the change in the rRNA 2’-O-methylation profile may have a diagnostic value. Therefore, the development of novel approaches for the analysis of rRNA and other long cellular RNA structures that enable a quantitative evaluation of the modification level of certain nucleotides in such RNAs is of great interest.

The current paper presents an adaptation of the method for the determination of 2’-O-methylated nucleotides in RNA to the use of 5’-fluorescently labeled primers with further analysis of reverse transcription (RT) termination products on an automated DNA-analyzer. Using the proposed approach, we have analyzed the influence of synthetic box C/D RNAs on the changes in the 2’-O-methylation profile of rRNA in human cells. It has been established that transfection of human breast adenocarcinoma MCF-7 cells with 28S rRNA-directed synthetic box C/D RNAs leads to an increase in the 2’-O-methylation level of certain nucleotides within the target RNA.

## EXPERIMENTAL

**Analysis of human 18S and 28S rRNA 2’-O-methylation profiles**

Nucleotide numbers of human rRNAs are presented according to GenBank: U13369 (7935–12969 nt) for 28S rRNA and X03205 for 18S rRNA.

Reverse transcription was carried out with primers containing 5’-terminal [^32^P] or 5(6)-carboxyfluorescein (FAM) label: 18-2 –5’-TAATGATCCTTCCGCAGGTTC- 3’ (complementary to the region 1849–869 nt of 18S rRNA); FAM-28-2.2 –5’-ATTGGCTCCTCAGCCAAGCA- 3’ (4608–627 nt of 28S rRNA) and FAM- 4491 –5’- GACGGTCTAAACCCAGCTCA-3’ (4491–4510 nt of 28S rRNA). A reagent mixture containing 3.0 µg of total cellular RNA and 1.4 pmol of primer was incubated at 70°C for 3 min and cooled to 4°C; then reverse transcription buffer containing 50 mM KCl, 50 mM Tris-HCl (pH 8.3), 4 mM MgCl_2_, and10 mM DTT, and also 3 AU/µl of M-MLV reverse transcriptase (Biosan, Novosibirsk, Russia) was added to the solution. The mix of dNTP was added separately to a final concentration of 2.0, 1.0, 0.1, or 0.04 mM. The reaction mixture was incubated at 40°C for 2 h. The RT products were precipitated with 75% ethanol, dried, and dissolved in deionized water.

The sequencing of the 18S rRNA region was conducted using the method of reverse transcription in the presence of ddNTP. In order to do this, 3.0 µlg of total cellular RNA and 1.4 pmol of primer 18-2 were incubated at 70°C for 3 min and cooled to 4°C. One type of ddNTP was then added to the reaction mixture: ddATP to a final concentration of 5.0 µlM, ddCTP –2.5 µlM, ddGTP –5.0 µlM or dTTP –5.0 µlM. The final concentration of the dNTP corresponding to the type of ddNTP in the solution was 25 µlM; the concentration of the other dNTP –100 µlM. The obtained solution was mixed with reverse transcription buffer (see above), 2.0 mM MnCl2, 10 mM DTT and 3 AU/µll of M-MLV reverse transcriptase. The mixture was incubated at 40°C for 90 min.

**Analysis of the products of reverse transcription of human ribosomal RNA**

The separation of fluorescently labeled products of the reverse transcription of rRNA was performed on an ABI3100 Genetic Analyzer (Applied Biosystems, Genomics Core Facility, Siberian Branch of the Russian Academy of Sciences). The data were analyzed using Peak Scanner Software version 1.0 (Applied Biosystems, USA). The relative change in the yield of RT products was determined on the basis of the peak area normalized to the total area of the peaks with relative intensities changing within the limits of 20%. The presented data are the average results of at least three independent experiments.

**Synthesis of artificial box C/D RNAs**

The analogues of box C/D RNAs were obtained via *in vitro *transcription according to [18, 19]: 28A4518 (98 nt)

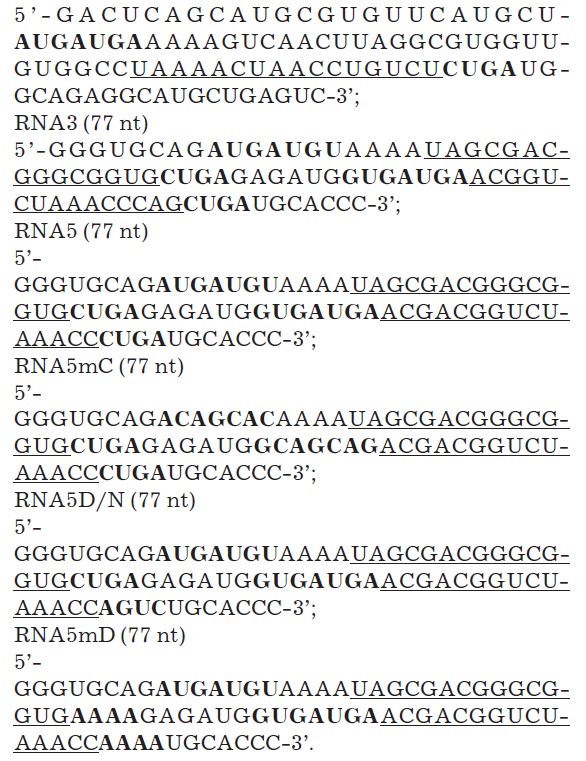



Ribosomal RNA recognition motifs are underlined; conserved elements (boxes C and D) are in bold.

**Transfection of MCF-7 cells with synthetic RNAs. Isolation of total cellular RNA**

MCF-7 cells (from the Russian cell culture collection of vertebrates, Institute of Cytology, Russian Academy of Sciences, St. Petersburg) were cultured in MDM medium with 10 mM L-glutamine and 40 μg/ml of gentamicin in the presence of 10% fetal bovine serum in 5% CO_2_ at 37°C.

Synthetic analogues of box C/D RNAs (10^-6^ M) were pre-incubated with Lipofectamine (Invitrogen, USA) at a concentration of 0.06 mg/ml at 20°C for 15 min. MCF- 7 cells were transfected with the RNA/lipofectamine complex (the final concentration of RNA in the medium was 7 × 10^-8^ M). Control cells were incubated in the medium with Lipofectamine (6 μg/ml) only. After 24 h of incubation, total cellular RNA was isolated using Trizol Reagent (Invitrogen, USA) according to the manufacturer’s protocol. The integrity of total RNA was assessed by electrophoresis using the Lab-on-chip platform for nucleic acid analysis (Agilent Bioanalyzer), and the samples with RIN values not less than 8.0 were further used in the experiments. The 2’-O-methylation profile of rRNA was analyzed as described above.

## RESULTS AND DISCUSSION

**Modification of the method of reverse transcription termination**

**Fig. 2:**
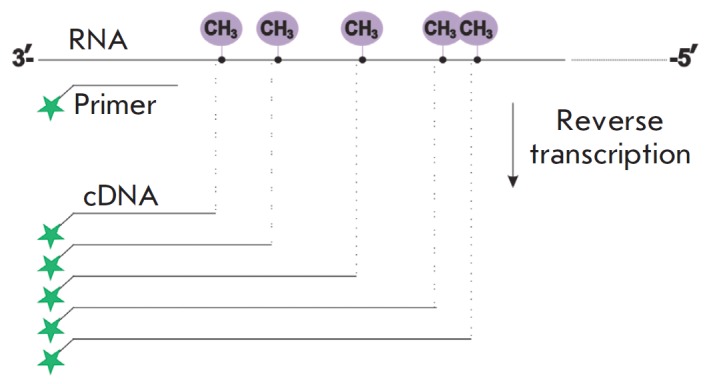
Method of reverse transcription termination

The method of reverse transcription termination is based on the ability of 2’-O-methylated nucleotides to cause the arrest of reverse transcriptase at dNTP concentrations less than 1 mM [20]. The location of 2’-O-methylated nucleotides in the analyzed RNA can be excluded from the length of RT termination products [21] (*Fig. 2*). Unlike the conventional technique, which assumes the reaction of reverse transcription with the radioactively labeled primer and separation of cDNA products in a polyacrylamide gel with further autoradiography, the approach we have developed is based on the use of fluorescently labeled primers and analysis of cDNA by capillary gel electrophoresis on an automated genetic analyzer.

**Fig. 3:**
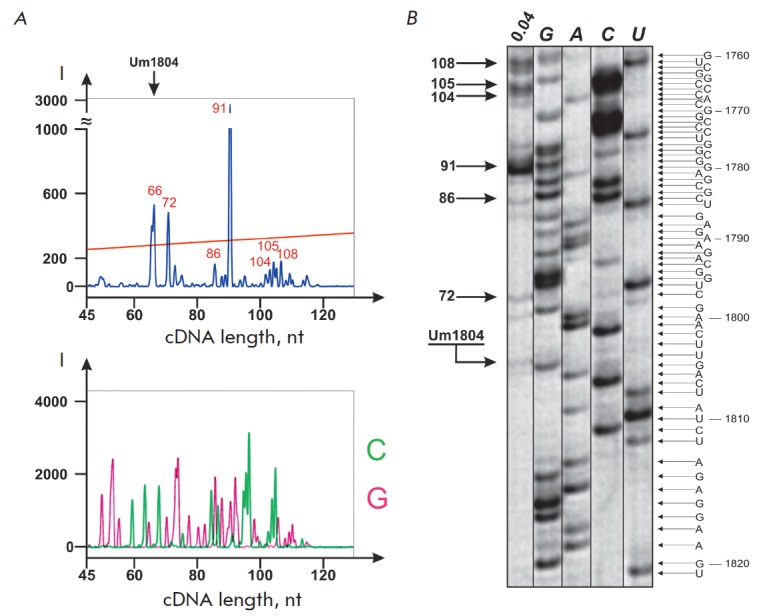
Comparison of the products of 18S rRNA reverse transcription termination with 5’-[^32^P]-labeled or 5’-FAM-labeled primer 18-2 (0.04 mM dNTP). *A *– 5’-FAM-labeled products of RT termination separated by capillary gel electrophoresis on an automated DNA analyzer (*upper insertion*); products of 18S rRNA RT with ddGTP and ddCTP (*lower insertion*). *B* – 5’-[^32^P]-labeled products of RT separated on 12% denaturing PAGE. Lane *0.04 *– products of RT termination at 0.04 mM dNTP. Lanes *G*,* A*, *C *and *U *–RT of 18S rRNA with ddCTP, dTTP, ddGTP and ddATP, respectively

The possibility of applying such an approach in the determination of 2’-O-methylated sites in RNA has been studied by conducting the reverse transcription of 18S and 28S rRNA from MCF-7 cells with [5’-^32^P]- or 5’-FAM-labeled primers. *Fig. 3* depicts the comparison between the typical results of the conventional method utilizing radioactively labeled primers and the method adapted to the analysis of fluorescently labeled cDNA products on an automated genetic analyzer. It can be seen from *Fig. 3 A,B* that the sets of cDNA products are in good agreement with each other in length and relative yield.

In order to determine precisely whether there is a correspondence between the detected products of RT termination and the location of specific nucleotides in the RNA template, we separated Sanger sequencing products of the 18S rRNA region (*Fig. 3A, lower insertion*).

Reproducibility of the results was assessed by using the data of three independent experiments of total cellular RNA reverse transcription with primers specific to various regions of 18S and 28S rRNAs, followed by separation of the cDNA products on a genetic analyzer. It has been established in a series of experiments that the main products of RT termination of the same regions of the rRNA template correspond to each other in the retention time (± 1 nt) and intensities of cDNA signals (± 10%) (data not shown).

**Fig. 4:**
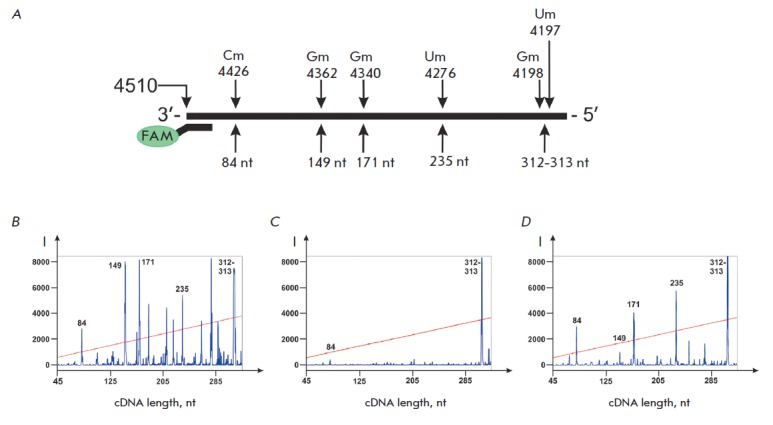
Analysis of cDNA products corresponding to the native sites of 2’-O-methylation in 28S rRNA. *A *– 2’-O-methylated nucleotides in 28S rRNA and lengths of corresponding termination products of RT with primer FAM-4491. *B–D *– analysis of FAM-labeled products of RT termination separated on an automated DNA analyzer. RT of 28S rRNA isolated from MCF-7 cells was conducted with the following concentrations of dNTP in the reaction mixture: *B *– 0.1 mM, *C *– 1.0 mM, *D *– 2.0 mM

Using the proposed method, we analyzed the sets of human 28S rRNA RT termination products at different concentrations of monomers in the reaction mixture. In addition to conventional conditions of the RT experiment that utilizes low dNTP concentrations (0.001–0.1 mM) in the reaction mixture [21], we also conducted the reaction at a high concentration of monomers (2.0 mM). *Fig. 4* demonstrates that decreased dNTP concentrations in the reaction mixture lead to a higher yield of cDNA products 84, 149, 171, and 235 nt in length that correspond to known sites of 2’-O-methylation: Cm4426, Gm4362, Gm4340, and Um4276, respectively *(Fig. 4 B,C)*. The reverse transcriptase terminates directly at a modified nucleotide (Gm4362, Gm4340 and Um4276) or at the 3’ neighboring nucleotide to the 2’-O-methylated (Cm4426). Moreover, it has been established that specificity of termination at 2’-O- methylated nucleotides can be achieved upon increasing dNTP concentration to 2.0 mM. For instance, at 2.0 mM of dNTP, the yield of the termination products that do not correspond to the 2’-O-methylated nucleotides of the RNA template decreases substantially compared to a reaction conducted at 0.1 mM dNTP (*Fig. 4 B,D*).

The advantage of the new approach is the possibility of quantitatively assessing the yield of RT termination products. Furthermore, the separation of termination products on an automated genetic analyzer enables to obtain more complete information on the location of termination sites in the RNA template due to the analysis of longer RNA regions compared to the conventional separation of cDNA in denaturing PAGE. The proposed approach can be applied for the identification of modified nucleotides in native RNAs, verification of the location of modification sites in synthetic RNAs, and for solving other tasks related to the analysis of the structures of long RNA molecules by the reverse transcription method as well.

**The influence of box C/D RNA analogues on the profile of 2’-O- methylation in human rRNA**

**Fig. 5:**
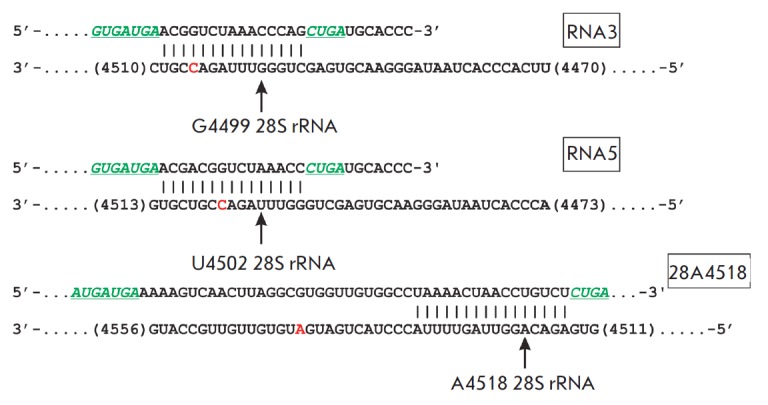
Design of the artificial analogues of box C/D RNAs. Conserved elements of snoRNA analogues – boxes C (C’) and D (D’) – are colored in green. Target nucleotides in rRNA are arrowed with the number of a nucleotide denoted according to [26]. Known sites of 2’-O-methylation in 28S rRNA are colored in red. The rectangles contain the designation of box C/D RNA analogues complementary to a specific region in the corresponding rRNA

The approach we have proposed can be used to study the influence of small nucleolar box C/D RNA analogues on the processing of target rRNAs in human cells. Earlier, J. Cavaille *et al. *demonstrated the possibility of directing 2’-O- methylation of rRNA using DNA constructs encoding box C/D RNAs [22]. We have designed and obtained synthetic analogues of human small nucleolar box C/D RNAs containing altered guide sequences (*Fig. 1*). We chose human rRNA nucleotides located within or in close vicinity to ribosomal PTC: G4499, U4502 and A4518 of 28S rRNA [23-25] as the targets for artificial box C/D RNAs. The analogues of small nucleolar box C/D RNAs were constructed to contain all the necessary components for directing 2’-O-methylation in a defined target nucleotide: conserved regions of boxes C and D (snoRNP protein recognition domains) and a sequence complementary to a region within the target rRNA (*Fig. 5*).

**Fig. 6:**
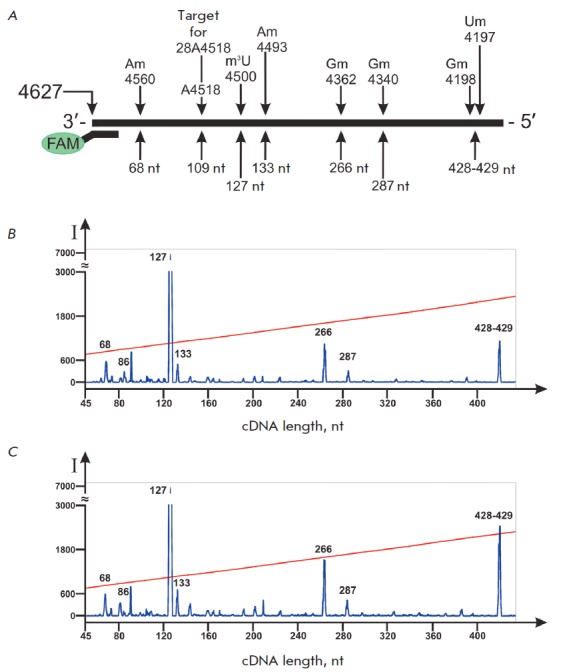
Influence of artificial box C/D RNA analogues on the yield of rRNA RT termination products. *A *– 2’-O-methylated nucleotides in 28S rRNA and lengths of corresponding termination products of RT with primer FAM-28-2.2. RT products of rRNA isolated from control MCF-7 cells (*B*) and from MCF-7 cells transfected with analogue 28A4518 (*C*). RT was conducted at 0.04 mM dNTP

The box C/D RNA analogue named 28A4518 has a structure appropriate for guiding 2’-O-methylation of A4518 of 28S rRNA. *Fig. 6 * demonstrates that transfection of RNA 28A4518 into human MCF-7 cells does not lead to the formation of a new termination product, 109-nt-long cDNA, corresponding to the target nucleotide. The absence of a nucleotide modification in the target rRNA has been discussed by us previously [18]. In particular, we assume that the observed absence of target nucleotide 2’-O-methylation may be an indication of the low content of the modified rRNA in cells due to its robust degradation. As shown in the studies by B. Liu and M.J. Fournier *et al.*, 2’-O-methylation of several nucleotides that comprise ribosome active centers in yeast induces degradation of target rRNAs and impedes cell growth [23, 27, 28].

Despite the absence of termination at the target nucleotide for RNA 28A4518, the transfection of cells with the synthetic analogue led to changes in the level of 2’-O-methylation of known sites of modification. *Fig. 6 B,C* demonstrates the increase in the yield of termination products that correspond to 2’-O-methylated Gm4362, Gm4198, and Um4197. A comparison of the cDNA product intensities has shown that the transfection of cells with RNA 28A4518 causes a 1.5-fold increase in the termination efficacy at Gm4362, while the yield of the 428-429-nt-long product (Gm4198 and Um4197) increases 2.2 times. The increase in the yield of RT products corresponding to the arrest of reverse transcriptase at 2’-O-methylated nucleotides indicates a higher modification level of these nucleotides in transfected cells.

A change in the modification level of rRNA nucleotides can be associated with an increase in the local concentration of the components of the complexes that perform 2’-O-methylation of nucleotides during the maturation of the rRNA nucleolar precursor (prerRNA). Gm4362, Gm4198, and Um4197 nucleotides of 28S rRNA are located at a considerable distance (more than 150 nt away) from the sequence complementary to the box C/D RNA (*Fig. 5*). An increase in the 2’-Omethylation level of the mentioned nucleotides can be associated with their spatial proximity in the structure of pre-rRNA with the nucleotide targets of box C/D RNA and migration of the methyltransferase complex components from the box-C/D-RNA/rRNA duplex to the nearest rRNA nucleotides. The possible influence of synthetic RNAs on the secondary structure of rRNA and interaction with independent trans-factors, such as helicases, that control the stages of rRNA maturation in eukaryotic cells, also cannot be excluded [29].

**Fig. 7:**
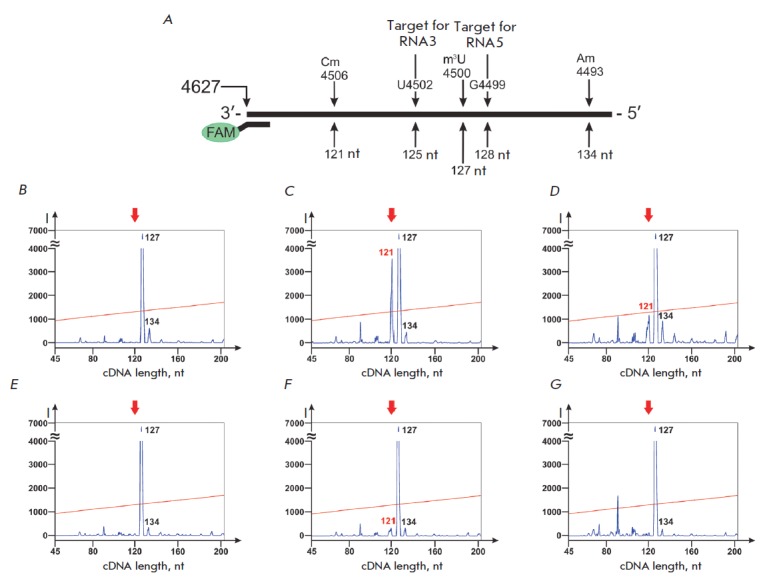
Influence of artificial box C/D RNA analogues on the 2’-O-methylation level of Cm4506 of 28S rRNA in human cells. *A – *location of primer FAM-28-2.2, 2’-O-methylated nucleotides in 28S rRNA and target nucleotides for box C/D RNA analogues. *(B–G) – *5’-FAM-labeled products of 28S rRNA RT termination ( 0.04 mM dNTP) isolated from control MCF-7 cells *(B) *and MCF-7 cells transfected with RNA3 *(C)*, RNA5 *(D)*, RNA5mC *(E)*, RNA5D/N *(F), *and RNA5mD* (G)*. Red arrow points to the location of the RT termination product that corresponds to Cm4506 of 28S rRNA. The127- nt-long product of RT termination corresponds to m3U4500 of 28S rRNA

We have also observed an increase in RT termination at known sites of 2’-O-methylation when studying the influence of other box C/D RNA analogues on human cells. In particular, the analogues RNA3 and RNA5 have been designed to direct 2’-O-methylation of G4499 and U4502 of 28S rRNA, respectively. The transfection of MCF-7 cells with RNA3 or RNA5 did not induce the modification of the target nucleotide but enhanced the 2’-O-methylation level of Cm4506 of 28S rRNA *(Fig. 7 C,D)*. The data shown in *Fig. 7* and *Table* indicate a 40- and 7-fold increase in the yield of the 121-nt-long cDNA product after transfection of MCF-7 cells with synthetic RNA3 and RNA5, respectively. This cDNA product forms as a result of M-MLV revertase termination at 2’-O-methylated Cm4506 of 28S rRNA during the elongation of primer FAM-28-2.2. The detected increase in the yield of the termination product indicates an enhanced 2’-O-methylation level of Cm4506.

In order to determine the structural features of box C/D RNA analogues that influence the level of native 2’-O-methylation of rRNA, we obtained the analogues of box C/D RNA5 with substitutions in box C(C’) or D(D’) sequences: RNA5mC, RNA5D/N, and RNA5mD. All the listed analogues had the same guide sequence targeted to U4502 of 28S rRNA (*Fig. 5*, *Table*). In RNA5mC, the sequences of boxes C (AUGAUGU) and C’ (GUGAUGA) had been substituted with (ACAGCAC) and (GCAGCAG), respectively. RNA5D/N contained the only substitution in box D structure (AGUC), while RNA5mC had both the D and D’ sequences (CUGA) changed to (AAAA).

It has been established that the substitutions in the structures of boxes D and C substantially decrease the efficacy of box C/D RNA analogue action. For instance, a substitution of only the box D sequence in the structure of RNA5 led to a 2-fold decrease in the relative yield of the termination product, while simultaneous substitution of both D and D’ boxes or both the C and C’ boxes decreased the yield to the level of the control cells *Fig. 7D*, *Table*. The obtained data led us to assume that boxes C and D are the key elements in the structure of artificial box C/D RNAs that influence the level of rRNA 2’-O-methylation when transfected into human cells. A single pair of boxes C/D in the structure of synthetic RNA is sufficient to influence rRNA modification. Elimination of the functional elements –boxes C and D –abolishes the action of snoRNA analogues on the structure of rRNA in transfected cells (*Fig. 7*, *Table*).

**Table:** Yields of the RT termination product corresponding to Cm4506 of 28S rRNA in MCF-7 cells transfected with box C/D RNA analogues

Name of box C/D RNA analogue	Alteration in box C/D RNA structure compared to RNA5	Yield of the RT termination product corresponding to Cm4506 28S rRNA**	Relative yield of the RT termination product ***, %
RNA5	-	1100 ± 154	100
RNA3	3-nucleotide shift to the 5’-end of rRNA in the complementary region	6500 ± 780	590
RNA5mC	box C (AUGAUGU) → (ACAGCAC); box C’ (GUGAUGA) → (GCAGCAG)	170 ± 25	15
RNA5D/N	box D (CUGA) → (AGUC)	570 ± 86	50
RNA5mD	Boxes D and D’ (CUGA) → (AAAA)	150 ± 25	13
Control*	-	150 ± 25	13

* Control cells were incubated with lipofectamine alone.

** Values of peak areas normalized to the total area of the peaks of RT termination products (±SD).

*** Relative yields of the RT termination product corresponding to Cm4506 28S rRNA compared to the yield of the same product of the RT of 28S rRNA isolated from MCF-7 cells transfected with RNA5.

The obtained data enabled us to conclude that box C/D RNA analogues influence post-transcriptional processing of ribosomal RNAs when penetrating into human cells. Despite the fact that transfection of box C/D RNA analogues into MCF-7 cells did not cause *de novo *2’-O-methylation of rRNA target nucleotides, the analogues enhanced the level of native 2’-O-methylation in 28S rRNA.

It has been recently shown that in a series of tumor cell lines an increase in the level of several box C/D RNAs is accompanied by an increase in fibrillarin, the key component of the nucleolar methyltransferase complex [12]. Moreover, the 2’-O-methylation profile of rRNA can vary in cancer cells of different origins [14]. In this regard, it is suggested that the regulation of ribosomal RNA maturation can affect oncogenic transformation of cells and be implicated in the control of cancer cell life cycle [30, 31]. Therefore, the observed effect of synthetic RNAs on 2’-O-methylation of rRNAs in human cells sheds light on perspectives for developing systems for the regulation of rRNA posttranscriptional maturation that can lay the groundwork for the development of novel therapeutic approaches. The proposed approach for the analysis of 2’-O-methylated nucleotides in RNAs can be used both to detect artificially directed RNA modifications and search for diagnostically significant differences in the profile of RNA modifications in human cells.

## CONCLUSIONS

This paper presents an adaptation of the reverse transcription termination method for determination of the location of 2’-O-methylated nucleotides in RNA and analysis of 5’-fluorescently labeled cDNA products by capillary gel electrophoresis on an automated DNA-analyzer. Using the proposed approach, we have analyzed the influence of synthetic analogues of small nucleolar box C/D RNAs on the 2’-O-methylation profile of human 28S rRNA in MCF-7 cells. It has been demonstrated that the transfection of cells with synthetic analogues of box C/D RNAs leads to enhanced RT termination at known 2’-O-methylation sites of rRNAs, indicating a significant increase in the modification level of certain nucleotides within the target rRNAs. Moreover, the conserved elements in the structure of snoRNAs – boxes C and D – play the key role in the action of synthetic RNAs.

Today it is believed that post-transcriptional modifications of different nucleotides in rRNA are independent processes. For instance, directed 2’-O-methylation of unmodified nucleotides of rRNA does not affect the modification outcome of other nucleotides [28, 32]. Our data demonstrate that box C/D RNAs that contain a guide sequence targeted to a defined nucleotide and do not induce 2’-O-methylation of the target nucleotide can influence the modification level of other nucleotides of rRNA. It is possible that the effect we observed reflects the functioning of a novel, native mechanism of coherent regulation of rRNA post-transcriptional modification. The implementation of such a mechanism can help modulate the 2’-O-methylation level of some nucleotides while affecting the modification of others that are brought together in the spatial structure of the nucleolar precursor of rRNA.

## Abbreviations

rRNA: ribosomal RNA
PTC: peptidyl transferase center
snoRNA: small nucleolar RNA
snoRNP: small nucleolar ribonucleoprotein
FAM: 5(6)-carboxyfluorescein
M-MLV: Moloney murine leukemia virus
dNTP: deoxynucleoside triphosphates
RT: reverse transcription
PAGE: polyacrylamide gel electrophoresis
pre-rRNA: precursor of ribosomal RNA

## References

[1] Lapeyre B. (2005). Top. Curr. Genet..

[2] Baxter-Roshek J.L., Petrov A.N., Dinman J.D. (2007). PLoS One..

[3] Piekna-Przybylska D., Decatur W.A., Fournier M.J. (2008). Nucleic Acids Research.

[4] Decatur W.A., Fournier M.J. (2002). Trends Biochem. Sci..

[5] Liang X.H., Liu Q., Fournier M.J. (2007). Molecular Cell.

[6] Esguerra J., Warringer J., Blomberg A. (2008). RNA..

[7] Liang X.H., Liu Q., Fournier M.J. (2009). RNA..

[8] Song X., Nazar R.N. (2002). FEBS Lett..

[9] Kiss-Laszlo Z., Henry Y., Bachellerie J.P., Caizergues-Ferrer M., Kiss T. (1996). Cell..

[10] Makarova J.A., Kramerov D.A. (2007). Molekulyarnaya Biologiya (Mosk.)..

[11] Reichow S.L., Hamma T., Ferre´-D’Amare´ A.R., Varani G. (2007). Nucleic Acids Research.

[12] Su H., Xu T., Ganapathy S., Shadfan M., Long M., Huang T.H., Thompson I., Yuan Z.M. (2014). Oncogene..

[13] Marcel V., Ghayad S.E., Belin S., Therizols G., Morel A.P. (2013). Cell..

[14] Belin S., Beghin A., Solano-Gonzalez E., Bezin L., Brunet-Manquat S. (2009). PLoS One..

[15] Teittinen K.J., Laiho A., Uusimaki A., Pursiheimo J.P., Gyenesei A., Lohi O. (2013). Cell. Oncol. (Dordr)..

[16] Montanaro L., Trere D., Derenzini M. (2008). Am. J. Pathol..

[17] Freed E.F., Bleichert F., Dutca L.M., Baserga S.J. (2010). Mol. Biosyst..

[18] Stepanov G.A., Semenov D.V., Kuligina E.V., Koval O.A., Rabinov I.V., Kit Y.Y., Richter V.A. (2012). Acta Naturae..

[19] Stepanov G.A., Semenov D.V., Savelyeva A.V., Koval O.A., Kuligina E.V., Rabinov I.V., Richter V.A. (2013). Biomed. Res. Int. ID:656158.

[20] Maden B.E., Corbett M.E., Heeney P.A., Pugh K., Ajuh P.M. (1995). Biochimie..

[21] Maden B.E. (2001). Methods..

[22] Cavaille J., Nicoloso M., Bachellerie J.P. (1996). Nature.

[23] Liu B., Fournier M.J. (2004). RNA..

[24] Graifer D., Molotkov M., Styazhkina V., Demeshkina N., Bulygin K., Eremina A., Ivanov A., Laletina E., Venyaminova A., Karpova G. (2004). Nucleic Acids Research.

[25] Bulygin K., Malygin A., Hountondji C., Graifer D., Karpova G. (2013). Biochimie..

[26] Lestrade L., Weber M.J. (2006). Nucleic Acids Research.

[27] Liu B., Ni J., Fournier M. J. (2001). Methods..

[28] Liu B., Liang X.H., Piekna-Przybylska D., Liu Q., Fournier M.J. (2008). RNA Biol..

[29] Rodriguez-Galan O., Garcia-Gomez J.J., de la Cruz J. (2013). Biochim. Biophys. Acta..

[30] Williams G.T., Farzaneh F. (2012). Nat. Rev. Cancer..

[31] Mannoor K., Liao J., Jiang F. (2012). Biochim. Biophys. Acta..

[32] Qu G., van Nues R.W., Watkins N.J., Maxwell E.S. (2011). Mol. Cell. Biol..

